# Recycled Aggregate Concrete: Effect of Supplementary Cementitious Materials and Potential for Supporting Sustainable Construction

**DOI:** 10.3390/ma18225183

**Published:** 2025-11-14

**Authors:** Yara Mouna, Benny Suryanto

**Affiliations:** 1 Institute of Sustainable Built Environment, Heriot-Watt University Dubai, Dubai Knowledge Park, Dubai P.O. Box 501745, United Arab Emirates; ym41@hw.ac.uk; 2 Institute of Sustainable Built Environment, Heriot-Watt University, Edinburgh EH14 4AS, Scotland, UK

**Keywords:** concrete, construction waste, embodied carbon, recycled aggregate, GGBS, silica fume, sustainability

## Abstract

Recycled aggregate sourced from construction and demolition waste presents a viable means of reducing the environmental impact associated with concrete production. However, previous research has shown that concrete incorporating recycled aggregate typically exhibits reduced strength and increased susceptibility to deterioration. In this work, eight concrete mixes were prepared using both virgin and locally sourced recycled coarse aggregate from the United Arab Emirates, with selected mixes incorporating various combinations of supplementary cementitious materials (SCMs) (ground granulated blast-furnace slag (GGBS) and silica fume). The mixes were tested over a period of 180 days to evaluate key mechanical properties, durability, and embodied carbon. It was found that partial replacement of Portland cement with GGBS and silica fume had no marked beneficial effect on the strength and water absorption of recycled aggregate concrete when compared to mixes containing virgin aggregate. However, improvements in resistance to chloride ingress and reductions in drying shrinkage were observed. Notably, the incorporation of SCMs resulted in a significant reduction in embodied carbon, with reductions in excess of 40% when compared with conventional Portland cement concrete.

## 1. Introduction

The global generation of Construction and Demolition (C&D) waste has now exceeded 1.1 billion tonnes annually, with its rapid increase over recent decades raising significant environmental concerns. These concerns are primarily driven by increasing demand for landfill space and the broader environmental impacts associated with C&D waste disposal [1,2]. In 2020 alone, the European Union generated approximately 300 million tonnes of C&D, accounting for approximately 15% of total waste production [3]. This figure is expected to increase further in the coming years, positioning C&D waste as one of the largest waste streams for the foreseeable future. In response to this growing challenge, the EU Waste Framework Directive has mandated that 70% of non-hazardous C&D waste be recycled [4].

The rapid growth of C&D waste generation is not exclusive to Europe and is evident in many countries around the world, particularly those experiencing construction boom in recent decades, such as the United Arab Emirates (UAE). In this region, C&D waste constitutes a significant portion of total solid waste. Estimates suggest that C&D waste accounts for more than half of the UEA total waste output [5], with Abu Dhabi and Dubai each generating over 2 million tonnes every year [6,7]. As construction in this region continues to grow rapidly, local authorities have implemented regulatory measures aimed at diverting C&D waste from landfills. For instance, Dubai mandates that at least 50% of C&D waste generated during construction activities be recycled [8]. Across the UAE, a variety of other initiatives have been implemented to reduce C&D waste volume [8,9]. Abu Dhabi, for instance, achieved a reduction of over 60% in C&D waste within a decade [10]. These achievements have been facilitated by collaborative efforts between public and private sectors, leading to the establishment of dedicated C&D waste recycling facilities in Abu Dhabi [11] and Sharjah [12], amongst other. These facilities process millions of tonnes of C&D waste every year, converting it into recycled aggregates primarily used in road construction and other infrastructure projects across the emirates [13].

Currently, there is growing interest in the use of recycled aggregate in wider infrastructure applications and more demanding contexts, such as concrete production. This trend is driven by increasing aggregate demand, the shortage of natural aggregate resources, and mounting pressure on landfill sites due to rising C&D activities [13,14]. However, several barriers hinder their widespread adoption. The main factors often cited are related to the properties and quality of recycled aggregates, which are influenced by the source and type of the raw materials and the processing methods employed [15,16]. Studies have shown that recycled aggregates typically exhibit inferior qualities compared to natural aggregates. This is primarily due to the presence of residual mortar adhered to the aggregate particles [17,18] and a weaker interfacial transition zone (ITZ) between the aggregate and the surrounding matrix. As a result, the incorporation of recycled aggregates in concrete often leads to reductions in both strength and durability [19]. Appropriate processing methods and technology are thus of importance to produce recycled aggregates with adequate and consistent quality [20,21].

In light of the growing emphasis on sustainable construction practices across the Middle East, the uptake of recycled aggregate concrete in the UAE remains relatively limited. Locally produced recycled aggregates are predominantly utilised in low-grade applications, such as road subbase and paving works, despite the substantial quantities of C&D waste generated across the region, which could otherwise be utilised as a valuable resource in sustainable concrete production. A primary research gap is identified in the lack of comprehensive studies evaluating the intrinsic properties of recycled aggregates sourced within the UAE, as well as the performance of concrete incorporating full replacement of natural coarse aggregates under UAE-specific environmental and material conditions. Moreover, there is a need to establish the optimum utilisation of SCMs to address the relatively low performance often associated with recycled aggregate concrete. To this end, this article presents a detailed experimental investigation into the mechanical, durability, and environmental performance of eight concrete mixes incorporating both virgin and locally produced recycled coarse aggregates sourced within the UAE. Particular emphasis is placed on assessing the influence of partially replacing Portland cement with locally sourced SCMs (GGBS and silica fume) on key concrete properties and embodied carbon. Specimens were cast and subjected to testing over a period of 180 days, with results evaluated against benchmark values reported in studies and the performance criteria specified in international standards. The work focuses on key mechanical properties, resistance to chloride ingress, and drying shrinkage.

## 2. Review of Recycled Aggregate and Concrete Properties

To provide the background to the work presented below, Table 1 presents a summary of the key physical and mechanical properties of recycled coarse aggregates compiled from over 150 studies published from 2003 to 2017 [16,18,19,21,22,23,24,25,26,27,28,29,30,31,32,33,34,35,36,37,38,39,40,41,42,43,44,45,46,47], alongside the permissible limits specified in a few international standards [48,49,50,51,52]. As can be seen, while most properties fall within acceptable ranges, water absorption and acid-soluble chloride content often exceed recommended thresholds. Compared to natural aggregates, recycled aggregates exhibit distinct differences in physical characteristics and durability. They tend to be more angular and exhibit fractured surfaces, resulting in a shape index ~34% higher than that of natural aggregates [21]. They also tend to display higher water absorption (up to 12%) than natural aggregates, which generally <4% [53]. Their resistance to abrasion is also lower, with Los Angeles abrasion values reaching 42%, compared to 30% for natural aggregates [54]. Sulphate content is generally higher (up to 0.3%, compared to <0.14% in natural aggregates [42,54]). These differences are largely attributed to the presence of adhered residual cement mortar, which contributes to increased porosity and reduced strength. The use of recycled aggregates in concrete may therefore compromise structural integrity, particularly when exposed to severe environmental exposure.

**Figure 1:**
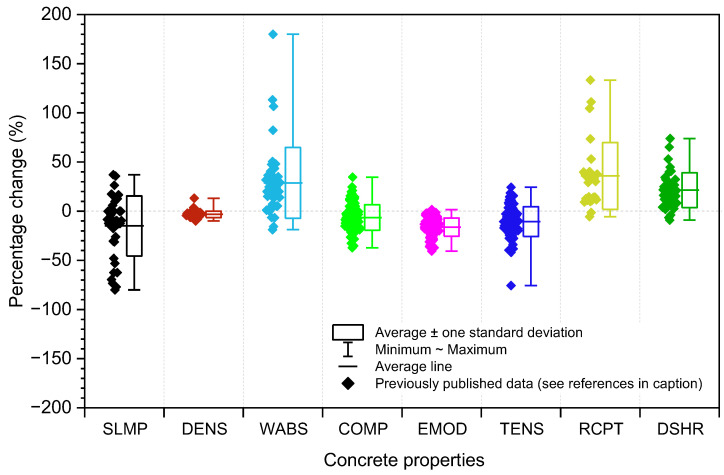
Effect of recycled coarse aggregate on various concrete properties according to previous studies [16,19,21,22,23,24,25,26,27,28,30,31,32,33,34,35,36,37,38,39,41,43,44,45,47,53,54,55,56,57,58,59,60,61,62,63,64,65]. Notes: SLMP: slump; DENS: density; WABS: water absorption; COMP: compressive strength; EMOD: elastic modulus; TENS: tensile strength; RCPT: rapid chloride penetration test; DSHR: drying shrinkage.

A considerable amount of work has been published on the properties of concrete containing recycled coarse aggregates derived from C&D waste [16,19,21,22,23,24,25,26,27,28,30,31,32,33,34,35,36,37,38,39,41,43,44,45,47,53,54,55,56,57,58,59,60,61,62,63,64,65]. Figure 1 provides a summary of the physical and mechanical properties of recycled aggregate concrete, including chloride penetration resistance and drying shrinkage, based on previous studies involving mixes with compressive strengths up to 60 MPa. It presents the percentage change in values relative to a reference mix incorporating virgin aggregate. While a wide variation in performance is evident, full replacement of virgin aggregate with recycled aggregate generally leads to reduced strength and durability, as indicated by higher water absorption and rapid chloride permeability tests (RCPT) values. These effects are primarily attributed to the presence of multiple ITZs [18], including remnants of old ITZ, which are typically weaker and more porous due to voids. This increased porosity compromises the overall performance of recycled aggregate concrete [66].

The partial replacement of Portland cement with supplementary cementitious materials (SCMs), such as ground granulated blast-furnace slag (GGBS) [67,68], fly-ash [67,69], and silica fume [68,70], has been shown to enhance the overall performance of recycled aggregate concrete. While higher GGBS replacement levels, particularly at 70%, were found to reduce early-age strength, moderate levels (50%) have yielded favourable outcomes [67]. Silica fume has proven effective in enhancing compressive strength and improving the aggregate/mortar interface due to its pozzolanic activity [69,71]. Similar improvements have also been reported for blends incorporating metakaolin and fly-ash [68], while the blend of fly ash with GGBS was found useful in improving the strength and durability of recycled aggregate concrete at extended curing periods. Apart from mechanical properties and durability, SCMs contribute to reduced cement content and lower embodied carbon [72,73]. Hence it is not surprising that SCMs are now routinely specified in concrete production to improve sustainability [74], including mixes containing recycled aggregates [74]. However, it is important to note that, as demonstrated by Jiménez and co-workers [54], the replacement of natural aggregates with recycled aggregates alone yields only modest reductions in carbon emissions (~3%), due to the dominant contribution of Portland cement to the overall carbon footprint.

## 3. Experimental Programme

### 3.1. Materials and Mix Proportions

The concrete mixes used within the experimental programme are presented in Table 2 and Table 3. The binders comprised Portland cement (PC) clinker CEM I 42.5N to BS EN 197-1:2011 [75]; CEM I cement blended with ground granulated blast-furnace slag to BS EN 15167-1:2006 [76] at a replacement level of 50% (hereinafter referred to as G50); CEM I cement blended with silica fume to BS EN 13263-1:2005 [77] at a replacement level of 10% (referred to as S10); and CEM I cement blended with 50% GGBS and 10% silica fume (referred to as G50S10). These replacement levels followed the recommendations in previous studies [68,69,70,71]. The oxide analysis of the cementitious materials is presented in Table 4.

**Figure 2:**
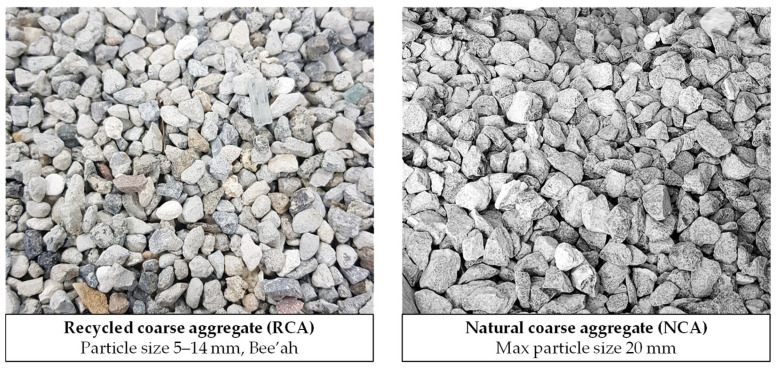
Photograph of recycled and natural coarse aggregates used in the work.

Two types of coarse aggregate were used: a natural, crushed aggregate (denoted NCA) comprising 20 and 10 mm limestone fractions blended in a 1.5:1 ratio [78] and a graded 5/14 recycled coarse aggregate (denoted RCA), sourced from a local C&D waste recycling company, Bee’ah (Sharjah, United Arab Emirates), see Figure 2. Although the RCA underwent a thorough processing procedure, traces of old cement mortar were still visible on its surface, albeit to a limited extent. A 70:30 blend of crushed rock fines with dune sand (sourced from the Ras Al-Khaimah (RAK) region) were used throughout. The properties of all aggregates used in the experiment are presented in Table 5.

With reference to the properties of the locally sourced RCA (Table 5), alongside the average values reported in previous studies (Table 1), it is evident that the local RCA satisfies most standard requirements and demonstrates performance comparable to findings from other studies. For instance, the specific gravity of the local RCA lies within the typical range reported (2.30–2.47) and exceeds the minimum standard requirement of 2.1. The water absorption value of 5.1% is well within the range reported in previous studies and remains significantly below the 7% limit specified in standards. Similarly, the flakiness and elongation indices for the local RCA are lower than both the values cited in the literature and the maximum permissible limits, indicating favourable particle shape characteristics. Moreover, the acid-soluble sulphate and chloride contents of the local RCA are considerably lower than values reported in the literature and remain well within acceptable limits. In terms of mechanical strength, the Los Angeles abrasion value of the local RCA lies within the 25–42% range reported in previous studies and complies with the less than 40% limit specified in standards, reflecting good aggregate strength characteristics. Overall, these results confirm that the locally sourced RCA is a suitable material for use in concrete applications.

### 3.2. Sample Preparation and Curing

Eight concrete mixes were prepared in accordance with the ACI 211 mix design procedure, each proportioned to achieve a target mean compressive strength of 50 MPa [79] and incorporating a constant free water/binder (w/b) ratio of 0.33. As summarised in Table 3, the first four mixes utilised NCA at 37% by volume, whereas the remaining four incorporated RCA at 30% by volume, adjusted to account for the differences in maximum aggregate size in compliance with the code requirement. A slightly higher quantity of fine aggregate was used in the RCA mixes to maintain a consistent binder volume of ~18% by volume across all mixes. In all mixes, a polycarboxylate high-range water reducer (PC400, conforming to Type G in ASTM C494 [80]) was added at a fixed dosage rate of 0.8% by weight of binder.

For each mix, a total of 42 specimens were cast to facilitate comprehensive mechanical and durability testing. This includes six 150 mm cubes for water absorption and porosity measurements; fifteen 150 mm cubes for density measurement and compressive strength testing; six 150 × 300 mm cylinders for elastic modulus and compressive strength evaluation; six, 100 × 100 × 500 mm prisms for flexural tensile strength measurements; six 150 mm cubes for rapid chloride permeability testing (RCPT); and three 50 × 50 × 200 mm prisms for drying shrinkage measurements.

Each concrete mix was manufactured in eight separate batches using a 65-litre pan mixer, following the procedures outlined in BS EN 12390-2:2019 [81]. The PC and SCMs were combined at the mixer alongside the aggregates. Immediately upon completion of mixing, the slump of the fresh concrete was determined in accordance with BS EN12350-2:2019 [82] under controlled laboratory environment (22 ± 3 °C). The fresh concrete was subsequently cast into steel moulds and compacted using a vibrating table. The specimens were then labelled and covered with plastic sheeting to minimise moisture loss during the initial curing phase. All specimens were demoulded after 24 h and transferred to a water-curing tank within the same laboratory environment (20 ± 10 °C and 40 ± 10% relative humidity) until required for testing.

### 3.3. Water Absorption, Porosity and Density

The rate of water absorption at 28 days was determined in accordance with BS 1881-122:2011 [83] using two 75 mm diameter cores extracted from 150 mm cubes. Each core was partially immersed to a depth of 50 ± 5 mm in a tray of water for 30 min, with the flat face placed at the bottom to ensure consistent exposure. Concrete porosity was determined from two additional 150 mm cubes by oven-drying to constant mass at 105 °C, followed by water immersion under a constant vacuum of 150 N/m^2^, in accordance with the procedure outlined in RILEM CPC11.3 [84]. The 28-day density of the concrete was measured in accordance with BS EN 12390-7:2019 [85], using the same 150 mm cubes employed for compressive strength testing.

### 3.4. Mechanical Property Testing

The strength development of the concrete over a 180-day period was determined on 150 mm cube specimens in accordance with BS EN 12390-3:2019 [81], using a 2000 kN compression testing machine. In addition, the elastic modulus and compressive strength were determined at 28 and 90 days on two 150 × 300 mm cylinders, following the procedures outlined in ASTM C469-02 [86]. On the same testing days, flexural tensile strength was evaluated using three notched prismatic beams (100 × 100 × 500 mm) subjected to four-point bending (4PBT), in accordance with UNI 11039-2:2003 [87].

### 3.5. Durability Testing

Durability performance was characterised through rapid chloride permeability tests (RCPT) at 28 and 90 days, following ASTM C1202-22 [88]. For each test, three 50 mm thick concrete disks were extracted from the central portion of 100 mm diameter core samples and placed in test cells containing 3% NaCl and 0.3 N NaOH solutions. The total electrical charge passed through each sample under a constant 60 V DC potential over a 6 h period was recorded to provide an indication of the concrete’s ability to resist chloride ion penetration. The laboratory temperature during testing was maintained at 20 ± 5 °C to minimise variability. Furthermore, samples were placed in a vacuum chamber to remove air from the pores within the concrete and then immersed in water for 24 h to ensure full saturation, in accordance with ASTM C1202-22.

Drying shrinkage was evaluated in accordance with BS 812-120 [89] by monitoring the longitudinal dimensional change in prismatic specimens, each with dimensions of 50 × 50 × 200 mm. Three specimens were tested per mix, with three sets of measurements taken on both sides of the sample and averaged to obtain representative values. The specimens were exposed to a natural environment (35 ± 10 °C and 60 ± 10% relative humidity) starting from 7 days after casting. Initial length measurements were recorded on the same day, followed by six subsequent readings over the remainder of the 180-day test period.

## 4. Results and Discussion

### 4.1. Slump of Fresh Concrete

The measured slump values for all concrete mixes are presented in Table 6. No significant variation was observed across the mixes when compared to the reference mix containing natural coarse aggregate and Portland cement (Mix N/PC), with slump values consistently falling within 200–220 mm range. These values correspond to the S4 classifications as defined in BS 8500-1:2015 [90] (i.e., >140 mm) and exceed the minimum workability threshold specified in ASTM C143-03 [91] (>100 mm). Given the high-range water-reducing admixture used in all mixes [44], no further enhancement in workability was observed with the inclusion of GGBS or silica fume, irrespective of aggregate type used.

### 4.2. Density, Porosity and Water Absorption of Concrete

Density values for all concrete mixes are presented in Table 6. As one would expect, the density of the recycled aggregate reference mix (R/PC) was lower than that of the virgin aggregate counterpart (N/PC), primarily due to the inherently lower density of recycled aggregates (see Table 5). This reduction is attributed to the presence of residual mortar on the recycled aggregate surface and the more porous nature of the recycled aggregate particles [45]. Moreover, the blended mixes incorporating GGBS and silica fume exhibited slightly lower densities (within 5%) than the reference mixes (Mixes N/PC and R/PC), which can be explained by the lower densities of these SCMs relative to Portland cement [92].

The porosity and cumulative water absorption values for all concretes mixes are presented in Table 6. It is evident that replacement of natural coarse aggregate with recycled coarse aggregate (i.e., transition from N to R series) resulted in a two to threefold increase in both porosity and water absorption regardless of the binder type. This increase is consistent with the higher water absorption capacity of recycled aggregates (see Table 5) and the higher overall porosity of the resulting concrete. The addition of GGBS and silica fume into the reference mix (Mixes N/G50, N/S10 and N/G50S10) resulted in a marked reduction in porosity and water absorption, particularly in mixes containing silica fume (i.e., reductions of approximately 50% were observed). The observed reduction is primarily due to the pozzolanic reaction and pore refinement resulting from the use of SCMs, which contributes to a denser microstructure and improved durability [68]. In contrast, the inclusion of GGBS and silica fume in the recycled aggregate mixes (Mixes R/G50, R/S10 and R/G50S10) did not yield significant improvements, with porosity and water absorption remaining comparable to the R/PC reference mix. While SCMs can be expected to improve the properties of the newly formed cement matrix, their effectiveness is limited by the presence of pre-existing porous old mortar adhered to the recycled coarse aggregates, particularly around the interfacial transition zone (ITZ). Remnants of the old ITZ [93] may also present, creating additional pathways for water ingress.

### 4.3. Compressive Strength of Concrete

Compressive strength results for NCA and RCA mixes are presented in Table 7 and Table 8, respectively. The values presented representing the mean of three specimens tested over 180 days (cubes) and 90 days (cylinders). The cylinder-to-cube compressive strength ratios at 28 and 90 days were 0.91 and 0.96, respectively, with a standard deviation of less than 3 MPa. Both ratios exceed the 0.82 ratio provided in BS EN 206:2013 [94] for a similar grade of concrete and fall within the range reported in previous studies using comparable specimen sizes and curing conditions (e.g., 0.88 for water cured specimens reported in [95]; 0.89 for recycled aggregate concretes in [96]; and 0.95 for concrete mixes manufactured from the same source of materials and tested using the same equipment [7]).

**Figure 3:**
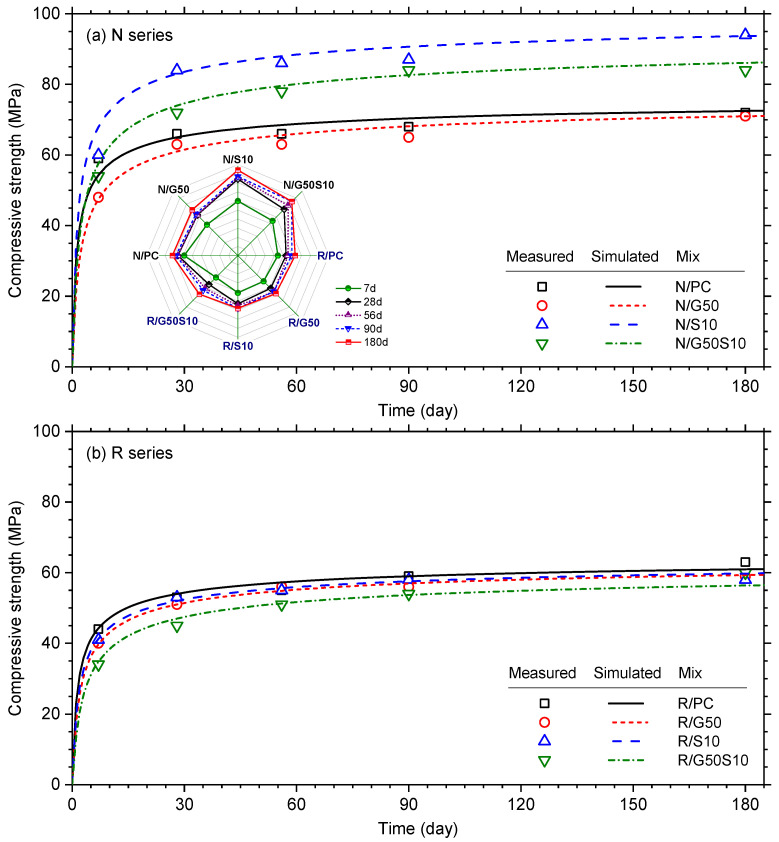
Cube compressive strength development for (**a**) N series and (**b**) R series mixes. Data points were taken from Table 7, while simulation curves generated using Equation (1).

To provide a better indication on strength development, Figure 3a,b present the compressive strength evolution for all virgin and recycled aggregate mixes over the 180-day test period. In these Figures, best fit curves are plotted in solid lines through the measurement data points using the exponential equation in BS EN 1992-1-1:2004 [97]:

(1) $$f\left(t\right)=f_{ref}e^{s(1-{\left(t_{ref}/t\right)}^{0.5})}\;\;\;\;\;\;\;\;MPa$$

where $f\left(t\right)$ is the predicted compressive strength at age t (day); $f_{ref}$ is the compressive strength at reference age $t_{ref}$ (= 28 days) and the exponent s is a constant indicative of strength development kinetics. Both $f_{ref}$ and s parameters are determined by fitting Equation (1) to the data points and presented in Table 9. In general, the values of the s parameter exhibit a broad range as expected, with higher s values indicating concrete with slower strength development, typically associated with pozzolanic reactions.

Concerning the two reference mixes, it is evident from Figure 3a,b that the R/PC mix consistently exhibited lower compressive strength than the N/PC mix, with a 13% reduction at 90 days. This is consistent with the 16% reduction observed in a recent experimental study using the same source of recycled coarse aggregate [7,98]. The influence of GGBS and silica fume is pronounced in the virgin coarse aggregate mixes (see Figure 3a). For instance, the inclusion of 50% GGBS (Mix N/G50) reduced the 7-day compressive strength by 18%, reflecting the slower hydration of the GGBS, as also reflected by the higher s value (see Table 9). By 180 days, however, N/G50 concrete achieved the same compressive strength to that of the N/PC mix, demonstrating the long-term benefits of GGBS [98,99]. In contrast, the addition of 10% silica fume resulted in strength enhancements of approximately 27% and 23% for Mixes N/S10 and N/G50S10, respectively (see Figure 3a), consistent with the range of values reported in previous studies [100,101].

**Figure 4:**
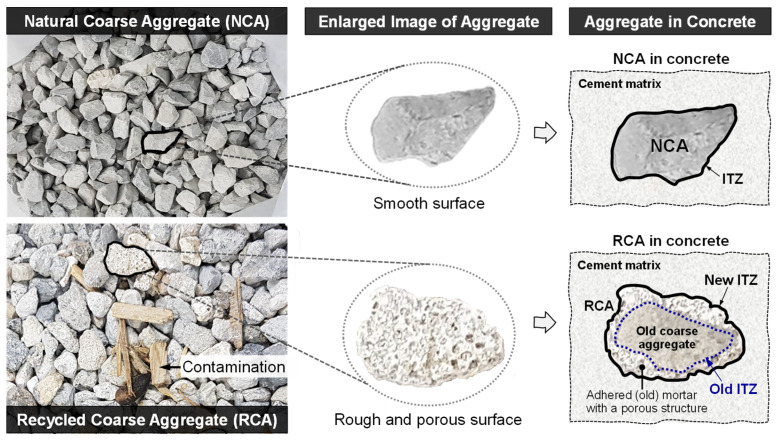
Schematic representation of the natural coarse aggregate (NCA) and recycled coarse aggregate (RCA) used in this study, together with an illustration of the differences in the ITZ for each aggregate type within the concrete. The NCA develops a new ITZ with the cement matrix, whereas the RCA forms a new ITZ with the cement matrix and retains an old ITZ associated with the adhered (old) mortar.

Conflicting evidence of the effect of GGBS and silica fume on concrete strength has been reported, with some reported enhancement in compressive strength [44,69] and others reported the contrary [70]. With reference to the results presented in this paper, it is apparent from Figure 3b that GGBS and silica fume had minimal impact on the compressive strength of recycled aggregate mixes, with all curves effectively superimposed on one another. This would indicate that the strength of this series of concrete mixes (R series) is largely insensitive to binder type. This is consistent with Al Martini et al. [92], who reported that SCM effects on compressive strength diminish when recycled aggregates are used. This strength limitation may stem from the inherent limited strength of the recycled aggregate or the compromised quality of the concrete due to the presence of old ITZ [102]. The influence of the new ITZ is likely limited, as it is dependent on the matrix strength and controlled by the binder type. Additionally, the reduced effectiveness of SCMs in these mixes may be attributed to the full replacement of virgin coarse aggregate with recycled aggregate. As illustrated in Figure 4, recycled aggregate has rougher surface with visible microcracks, in contrast to the smoother surface of natural aggregates. This surface irregularity can hinder effective bonding with the cement matrix, potentially leading to void formation around the aggregate particles [103]. These voids are often filled with fine particles or air, resulting in surface pores that the binder matrix cannot fully penetrate. As a result, continuous pathways may form, compromising the integrity of the matrix and increasing its permeability, regardless of any enhancements made to the binder [104,105].

The limited influence of SCMs on the compressive strength of the recycled aggregate concrete mixes could be attributed to the untreated nature of the recycled coarse aggregate used in this study, as previous studies have shown that treated recycled aggregates interact more effectively with SCMs [106]. Moreover, the limited effectiveness of SCMs in mixes containing recycled coarse aggregate (R series) might be attributed to the full replacement of virgin coarse aggregate with recycled aggregate [107].

### 4.4. Modulus of Elasticity and Flexural Tensile Strength

The elastic modulus values for all concrete mixes are presented in Table 10. In general, the results indicated that the modulus of elasticity for the recycled coarse aggregate reference mix (R/PC) is 27% lower than that of the corresponding natural coarse aggregate reference mix (N/PC) and falls within the range of reductions (up to 45%) reported in previous studies [18]. This reduction is attributed to the inherently lower stiffness of the recycled aggregate and the reduced volume fraction of coarse aggregate in the recycled mixes.

With respect to flexural tensile strength, it is evident from Table 10 that the replacement of NCA with RCA appears to have a less pronounced effect than that observed for compressive strength [7,108]. The R/PC mix exhibited flexural tensile strength values at both 28 and 90 days that were only approximately 10% lower than those of the virgin aggregate concrete reference mix (N/PC). The incorporation of GGBS and/or silica fume, which was expected to enhance the matrix quality around the aggregate particles and hence improve the tensile strength, did not result in significant enhancements in tensile strength across all virgin and recycled aggregate mixes. These findings align with those reported in previous studies [68,69].

#### 4.4.1. Prediction of Flexural Tensile Strength

The measured flexural tensile strengths for all mixes are compared with predictions from various design standards and empirical equations proposed in previous studies, particularly those developed for concrete containing recycled coarse aggregate [20,109] (see Table 11).

**Figure 5:**
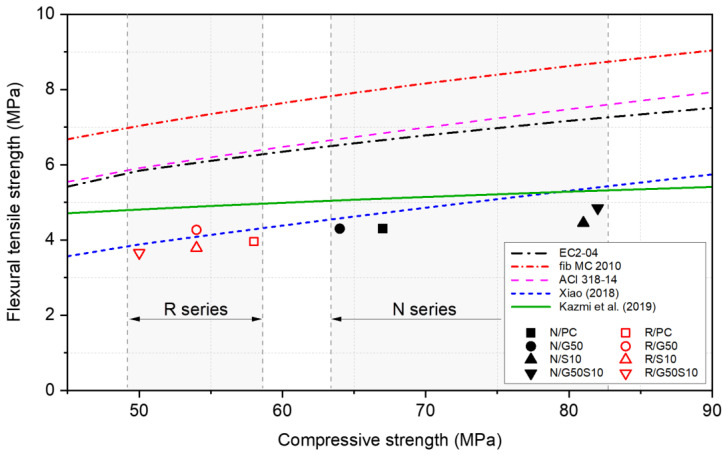
Comparison of predicted and observed flexural tensile strength.

Figure 5 presents the flexural tensile strength predictions from various standards and studies, plotted against concrete compressive strength. It is clear that the flexural strengths of all concrete mixes (N and R series) were significantly lower than the majority of the predictions provided by EC2-04 [97], ACI 318-14 [111], and *fib* Model Code 2010 [110]. With regard to the R series, it is interesting that the results obtained in this study align well with the predictions of the Xiao (2018) [20] equation (Equation (7)), with an average difference of only ~7%. This agreement is likely due to the inclusion of an additional parameter (i.e., the RCA replacement ratio) in the model. In contrast, the predictions from the Kazmi et al. (2019) [109] equation (Equation (8)), developed for a wide range of RCA replacement ratios (15–100%), tend to overestimate the measured flexural strengths, with an average deviation of ~11%.

### 4.5. Rapid Chloride Permeability Test

**Figure 6:**
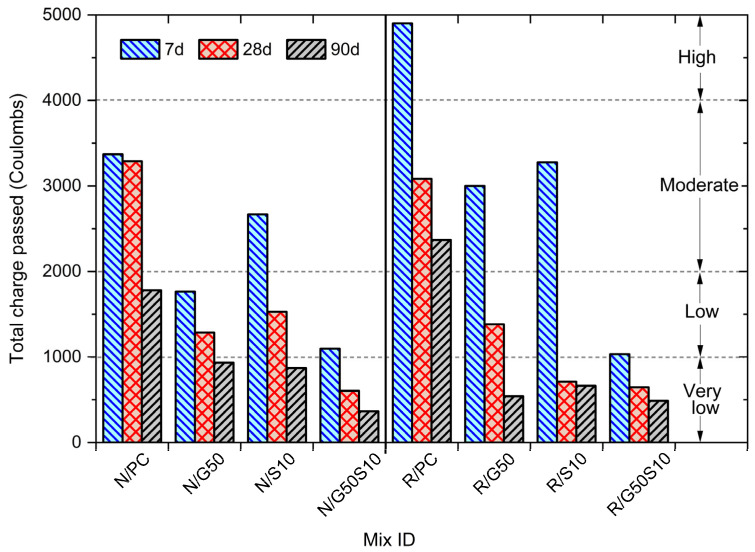
Measured total charge passed for the concrete mixes and comparison with chloride ion penetrability classification according to ASTM C1202 [88].

The total charge passed over a 6 h period for all concrete mixes at 7, 28, and 90 days is presented in Figure 6, together with the chloride ion permeability classification defined in ASTM 1202 [88]. As expected, there is a consistent reduction in chloride permeability with increasing curing time, reflecting continued hydration and refinement of the pore-structure [99,112]. At 90 days, the recycled aggregate concrete mix (R/PC) exhibited values over 30% higher than the reference mix containing virgin aggregate concrete (N/PC), which is in broad agreement with values reported in previous studies [21,113]. The most pronounced reduction occurred between 28 and 90 days, highlighting the advantages of continued hydration and pozzolanic reactions. The incorporation of GGBS and silica fume significantly improved resistance to chloride penetration over the longer term, particularly in ternary blended systems (Mixes N/G50S10 and R/G50S10). After 90 days, chloride ion penetrability for these mixes transitioned from “High” and “Moderate” classifications, as observed in N/PC and R/PC mixes, to “Very Low” [88] (see Figure 6), in accordance with ASTM 1202 classification. This highlights the enhanced pore structure in blended cements, resulting from ongoing hydration and pozzolanic activity [93,114]. It is noteworthy that the ternary blend containing recycled aggregates (R/G50S10), despite performing slightly below its reference mix (N/G50S10), exhibited superior resistance to chloride ingress compared to Mix N/PC. This highlights its potential for durable structural applications in aggressive chloride environments.

### 4.6. Drying Shrinkage

**Figure 7:**
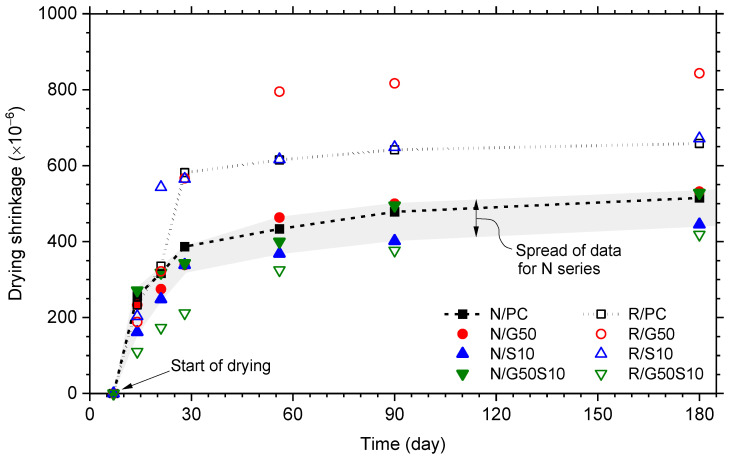
Development of drying shrinkage over time. Drying commenced 7 days after casting under a temperature of 35 ± 10 °C and RH of 60 ± 10%.

Drying shrinkage is an important factor to consider when it comes to durability as concrete with excessive shrinkage can lead to cracking in the cover region which, in turn, will accelerate the deterioration processes and hence impair the long-term durability [115]. The drying shrinkage values measured from all mixes over a 180-day period are presented in Figure 7, with drying commencing 7 days after casting. All mixes exhibited a rapid increase in drying shrinkage during the initial few weeks, followed by a more gradual increase throughout the remainder of the test duration. At 180 days, shrinkage value for the virgin aggregate (N) series ranged from 450 to 530 microns (shaded area), while the recycled aggregate (R) series displayed a much large spread in value, ranging from 420 to 840 microns, noticeably due to the high increase in value during the first few weeks of exposure (discussed below). The recycled aggregate reference (R/PC) mix exhibited shrinkage values approximately 30% higher than the virgin aggregate reference (N/PC) mix, consistent with the 60% increase reported in previous studies [106,114].

With regard to normal aggregate concrete containing GGBS (Mixes N/G50 and N/G50S10), it is evident from Figure 7 that initial shrinkage increased at a faster rate than those observed in the silica fume-only mix (N/SF10). This could be attributed to the slower hydration [44,115] and more porous nature of GGBS at early ages. Given the dominant role of moisture loss in drying shrinkage [115,116], higher early-age porosity must have contributed to the increased shrinkage observed. Similarly, the use of GGBS in recycled aggregate concrete (mix R/G50) resulted in nearly 30% increase in drying shrinkage at 90 days compared to the reference (R/PC) mix. The ternary blend (R/G50S10) displayed a more gradual shrinkage progression and achieved the lowest overall drying shrinkage values (e.g., approximately 23% and 70% lower than those of N/PC and R/PC mixes, respectively). Although this ternary mix had comparable total porosity to other recycled aggregate concrete mixes (see Table 6), the reduced drying shrinkage suggests that this mix must have a more disconnected pore network, which may limit moisture transport.

### 4.7. Equivalent Carbon Emissions

The greenhouse gas (GHG) emissions associated with each concrete mix were estimated based on cradle-to-gate data and expressed as the carbon dioxide equivalent per tonne of materials (kgCO_2_e/t). This assessment accounts not only for direct CO_2_ emissions but also for other environmentally detrimental gases, such as methane (CH_4_), nitrogen oxides (NO_x_), and sulphur oxides (SO_x_), which are emitted during raw materials extraction, energy consumption, and transportation activities. The total GHG emissions were calculated using the following equation [117]:

(9) $${GHG}_{Tot}=\sum_{i=1}^{n}m_{i}(d_{i}e_{i}+p_{i})$$

where $m_{i}$ is the mass of component *i* (kg); $d_{i}$ is the transportation distance (km); $e_{i}$ is the emission factor for the transportation mode (kgCO_2_e/(km.t)); and $p_{i}$ is the production-related emissions per unit mass of component *i* (kgCO_2_e/t). CO_2_e values for cement production have been reported to range from 576 to 1000 kgCO_2_e/t [117,118]_,_ depending on the manufacturing technology and fuel type. In this study, a representative value of 709 kgCO_2_e/t [118] was adopted. The corresponding estimated values for GGBS and silica fume were taken as 121 kgCO_2_e/t [117,119] and 96 kgCO_2_e/t [117,120], respectively, inclusive of overseas transportation to the UAE. For virgin aggregates, cradle-to-gate emissions were assumed to be 46 kgCO_2_e/t for coarse aggregate and 14 kgCO_2_e/t for fine aggregate [121,122]. Recycled aggregate emissions were reported in the range 16–63 kgCO_2_e/t, depending on the recycling method and location, with values less than 37 kgCO_2_e/t typically reported in the case of no heating treatment used in the recycling process [123]. Given that the supplier of the recycled coarse aggregate used in this study employed a conventional (mechanical) recycling method with relatively low energy consumption, a value of 22 kgCO_2_e/t was considered, consistent with previous studies using similar treatment method [54,123].

**Figure 8:**
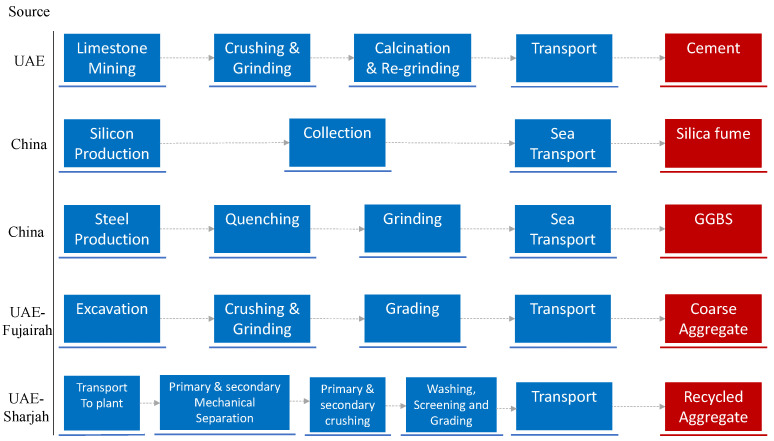
Schematic diagram of extraction, processing and transportation of main constituents of concrete studied.

**Figure 9:**
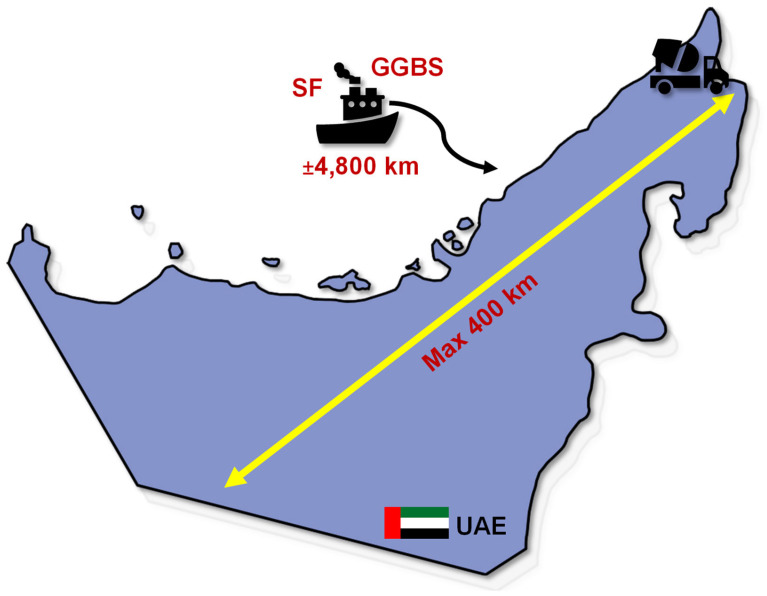
Schematic diagram illustrating transportation and the estimated maximum distance from the source to the construction sites within the UAE, assuming a worst-case in-country transportation distance of 400 km.

Table 12 presents the cradle-to-gate CO_2e_ values for each concrete component, and for completeness, includes the corresponding values taking into account transportation emissions from the point of manufacture within the UAE to the concrete plant (Figure 8). When estimating the transportation component, the analysis assumed a worst-case transportation distance of 400 km within the UAE (see Figure 9). Emissions factors for road and sea transports were taken as 0.09 and 0.02 kgCO_2_e/(km.t), respectively, to account for the import of GGBS and silica fume from overseas.

**Figure 10:**
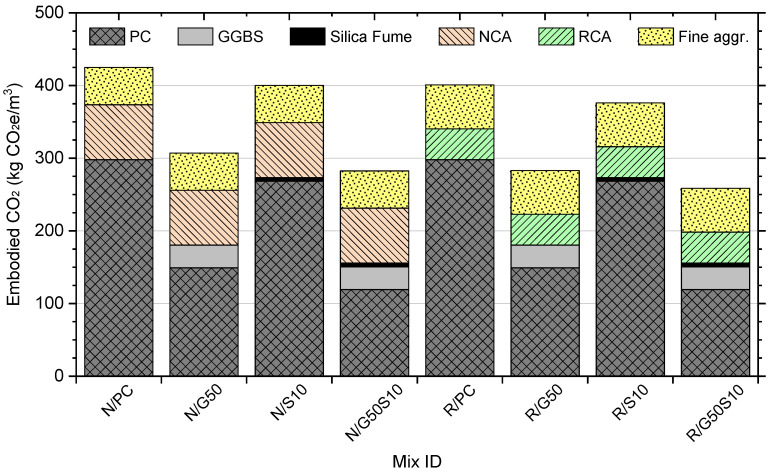
Embodied carbon of concrete mixes studied.

Figure 10 illustrates the total CO_2_e emissions for each concrete mix. It is evident that R/PC mix exhibited only a 6% reduction in CO_2_e emissions compared to the N/PC mix, indicating that the replacement of virgin coarse aggregate with recycled aggregate results only in marginal carbon savings. It also highlights the dominant influence of Portland cement content on overall CO_2_e emissions. The general trend is that CO_2_e emissions decrease with increasing SCM content due to the reduction in cement content. The greatest reduction was achieved in the ternary blended mix (R/G50S10), which incorporated 50% GGBS and 10% silica fume. This mix demonstrated a considerable 41% reduction in CO_2_e emissions relative to the control (N/PC) mix. According to the ICE concrete classification system for embodied carbon [124], the R/G50S10 mix transitions from Class F without SCMs (as observed in Mixes N/PC R/PC) to Class A with SCMs, highlighting its potential as a highly sustainable concrete material.

## 5. Influence of SCMs

**Figure 11:**
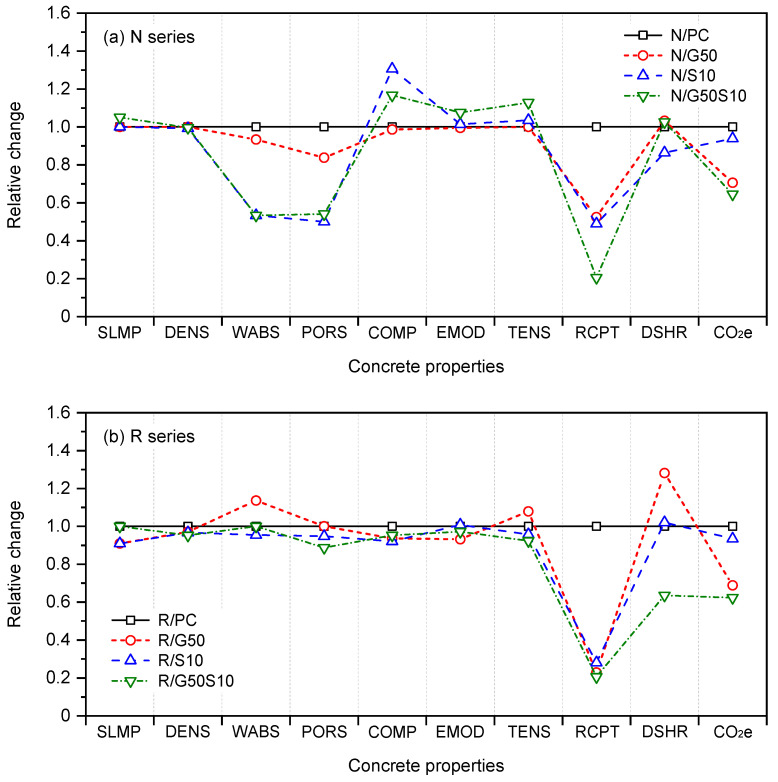
Summary of the mechanical and durability properties examined in the study: (**a**) for N series with natural aggregate; (**b**) for R series with recycled aggregate. Notes: SLMP: slump; DENS: density; WABS: water absorption; PORS: porosity; COMP: compressive strength; EMOD: Elastic modulus; TENS: flexural tensile strength; RCPT: rapid chloride penetration test; DSHR: drying shrinkage; CO_2_e: equivalent CO_2e_ emissions.

Figure 11a,b present the relative changes in key properties of concrete for mixes incorporating SCMs, for both normal aggregate concrete (N series) and recycled coarse aggregate concrete (R series). In these Figures, the relative changes were determined by normalising the measured values from mixes containing SCMs against the corresponding reference mixes containing only Portland cement (i.e., N/PC and R/PC).

Regarding the N series (Figure 11a), the partial replacement of Portland cement with GGBS and/or silica fume generally improved the concrete properties. Notable reductions in water absorption and porosity (up to 47%) were evident, attributed likely to the pozzolanic activity of the SCMs and the resulting refinement of the pore structure, particularly through reduced pore connectivity. Mechanical performance also improved, with compressive strength increasing by approximately 20% and flexural tensile strength by ~7% in mixes containing 10% silica fume (N/S10) and the ternary blend (N/G50S10). Chloride ion permeability was significantly reduced, with values over 50% lower than those of the reference mix (N/PC). Drying shrinkage was also reduced by up to 14%, particularly in the N/S10 mix (from 530 to 450 microns). However, replacing virgin aggregate with recycled aggregate had a significant impact on the properties of the concrete. This replacement led to reductions in compressive and tensile strengths by approximately 12% and 8%, respectively, while increasing water absorption and porosity by about 47% and 55%. In terms of durability, the use of recycled coarse aggregates also resulted in higher chloride permeability (33%) and drying shrinkage (28%). These changes indicate a general decline in performance compared with the reference PC mix made with virgin aggregates.

For the R series (Figure 11b), the partial replacement of Portland cement with GGBS and/or silica fume had limited influence on the mechanical properties of the recycled aggregate mixes. Compressive strength, modulus of elasticity, and flexural tensile strength remained largely unchanged across mixes incorporating GGBS and/or silica fume. However, durability related properties exhibited marked improvement. Chloride ion permeability reduced by up to 80%, and embodied carbon emissions decreased by approximately 40%, particularly in the ternary blend (R/G50S10). Drying shrinkage varied across mixes, with Mix R/G50 exhibited a 28% increase in value, while Mix R/S10 exhibited negligible change. The ternary blend (R/G50S10) demonstrated the most favourable shrinkage performance, with a 36% reduction in drying shrinkage relative to the R/PC mix. As note earlier, despite having comparable total porosity to other recycled aggregate concretes, the reduced shrinkage in R/G50S10 suggests a more disconnected pore network, likely contributing to improved dimensional stability.

Given the satisfactory mechanical performance, enhanced durability, and notable reduction in embodied carbon, the developed recycled aggregate concrete incorporating SCMs is considered suitable for durable and sustainable construction. Its application is particularly relevant to marine infrastructure and urban sustainability projects within the UAE, including both cast in situ and precast elements exposed to chloride-rich environments, where the ability to mitigate reinforcement corrosion is of considerable importance. While sulphate resistance was not assessed in this study, the mixes may also be suitable for foundations and general underground structures in mild exposure conditions. Although the developed mixes demonstrated promising performance, certain limitations must nevertheless be acknowledged. The recycled coarse aggregates used in this study were obtained from a single local supplier and employed without further treatment. As such, the influence of aggregate variability, source characteristics, and pretreatment on concrete performance remain unexplored. It is of the primary intention of this study to establish a baseline for future research, and subsequent investigations should therefore explore the performance of these mixes under specific conditions at the structural scale.

## 6. Concluding Remarks

Based on the comprehensive experimental programme undertaken on eight concrete mixes incorporating both virgin and recycled coarse aggregates, with and without supplementary cementitious materials (SCMs), the following conclusions can be drawn:

- The incorporation of recycled coarse aggregate resulted in measurable reductions in compressive strength, flexural tensile strength, and elastic modulus. It also led to increased water absorption, porosity, chloride ion permeability, and drying shrinkage, indicating a lower overall performance compared to the reference PC mix containing virgin aggregates and Portland cement as the main binder.
- The partial replacement of Portland cement with GGBS and/or silica fume significantly enhanced the physical and mechanical properties of concrete containing virgin coarse aggregates. Improvements were observed in compressive strength, tensile strength, elastic modulus, and reductions in porosity and water absorption. These enhancements are attributed to the pozzolanic activity of the SCMs and the resulting refinement of the concrete micropore structure.
- The inclusion of SCMs in recycled aggregate concrete did not yield notable improvements in mechanical properties. This is likely due to the predominant influence of residual mortar adhered to the recycled aggregate particles and the inherently higher porosity of the recycled aggregates. Nevertheless, the combined use of GGBS and silica fume (ternary blend) exhibited significant improvements, including up to 80% reduction in chloride permeability and 36% reduction in drying shrinkage.
- The recycled aggregates used in this study, sourced locally within the UAE, met the minimum requirements specified in various international standards and exhibited performance characteristics consistent with those reported in previous studies. Their increased use in general concreting applications should therefore be encouraged to promote sustainable development initiatives across the Middle East and beyond. The combined use of recycled aggregate and SCMs is particularly recommended for applications where long-term durability is of primary importance, such as in chloride-rich environments.
- The carbon emission of concrete is closely linked to the quantity of Portland cement used. The most significant reduction in CO_2_e emissions (exceeding 40%) was achieved through the combined use of 50% GGBS, 10% silica fume, and 100% recycled aggregate. This optimised mix elevated the sustainable classification of the concrete from Class F to Class A (Green) on the ICE embodied carbon ranking scale, underscoring its potential as a low-carbon, high-performance material for future infrastructure development.
- Owing to the comparable mechanical performance, significant durability enhancement from the ternary SCM blend, reduced embodied carbon, and improved shrinkage control, the developed recycled aggregate concrete mix is deemed suitable to support durable and sustainable construction practice in the UAE. Potential applications include both cast in situ and precast elements in marine-exposed infrastructure, where chloride-induced reinforcement corrosion is a major concern. The developed mix may also be applicable for general urban infrastructure, including foundations and other underground structures, subject to further validation under relevant exposure conditions.

Current studies are continuing to investigate the structural response of full-scale members made with 100% recycled coarse aggregate. Research is also continuing regarding assessing the long-term durability of recycled aggregate concrete under combined deterioration mechanisms, such as carbonation and corrosion [125]. Probabilistic approaches, including Life Cycle Assessment and Life Cycle Cost Analysis, will be applied to account for variability in material properties and exposure conditions, particularly in light of future climate change scenarios [126].

## Figures and Tables

**Table 1:** Comparative summary of recycled coarse aggregate properties reported in literature with specification limits in various standards.

Property	Values from Prior Studies [17,20,21,22,23,24,25,26,27,28,29,30,31,32,33,34,35,36,37,38,39,40,41,42,43,44,45,46,47,48]	Limits from Standards
Specific gravity	2.3–2.47	>2.1 [48]
Bulk density (compacted)	1.1–1.35	<2 [51]
Water absorption	3.1–12%	<7% [48]
Flakiness index	<17%	<35% [50]
Elongation index	<20%	<35% [50]
Acid soluble sulphate	<0.14%	<0.8% [48]
Acid soluble chloride	<0.3%	<0.05% [49]
LA abrasion value	25–42%	<40% [48]
Ten percent fines value (kN)	120–160	>80 [48]

**Table 2:** Mix description.

ID	Description
N/PC	Crushed limestone, normal coarse aggregate (NCA) concrete with plain Portland cement binder
N/G50	NCA concrete with 50% replacement of the cement with GGBS
N/S10	NCA concrete with 10% replacement of the cement with silica fume
N/G50S10	NCA concrete with 60% replacement of the cement with GGBS (50%) and silica fume (10%)
R/PC	Recycled coarse aggregate (RCA) concrete with plain Portland cement binder
R/G50	RCA concrete with 50% replacement of the cement with GGBS
R/S10	RCA concrete with 10% replacement of the cement with silica fume
R/G50S10	RCA concrete with 60% replacement of the cement with GGBS (50%) and silica fume (10%)

**Table 3:** Summary of concrete mixes (water/binder = 0.33).

Mix Designation	CEM I kg/m^3^	GGBS kg/m^3^	SF kg/m^3^	NCA 20 mm kg/m^3^	NCA 10 mm kg/m^3^	RCA 5/14 mm kg/m^3^	Fine <5 mm kg/m^3^	Dune Sand kg/m^3^
N/PC	400	–	–	553	368	–	718	308
N/G50	200	200	–	553	368	–	718	308
N/S10	360	–	40	553	368	–	718	308
N/G50S10	160	200	40	553	368	–	718	308
R/PC	400	–	–	–	–	735	846	362
R/G50	200	200	–	–	–	735	846	362
R/S10	360	–	40	–	–	735	846	362
R/G50S10	160	200	40	–	–	735	846	362

**Table 4:** Oxide analysis of cementitious materials used in the experimental programme.

% by Weight	CEM I	GGBS	SF
CaO	63.92	41.97	+
SiO_2_	20.09	31.23	90
Al_2_O_3_	4.75	12.75	+
Fe_2_O_3_	3.45	1.04	+
MgO	1.47	4.45	+
P_2_O_5_	0.02	0	+
Na_2_O	0.14	0.2	+
K_2_O	0.55	0.23	+
SO_3_	2.2	1.08	+
Clˉ	0.05	0.02	+
MnO	+	0.12	+
Mn_2_O_3_	+	0.27	+
TiO_2_	+	0.77	+
SrO	+	0.08	+
CaCO_3_	+	+	+
LOI	3.0	2.12	2.23
Origin	Dubai	China	China

Note: ‘+’ indicates not determined.

**Table 5:** Physical and mechanical properties of aggregate.

Properties	Unit	NCA 20 mm	NCA 10 mm	Fine < 5 mm	Dune Sand	RCA 5–14 mm	Recommended Values for RCA
SG (oven dry)		2.67	2.67	2.61	2.59	2.36	≥2.1 [48]
SG (SSD)		2.68	2.68	2.65	5.62	2.48	–
Apparent SG		2.70	2.71	2.72	2.68	2.69	–
Water absorption	%	0.5	0.5	1.6	1.2	5.1	≤7 [48]
Bulk density (compacted)	×10^3^ kg/m^3^	1.50	1.49	1.51	1.66	1.35	≤2 [51]
Bulk density (uncompacted)	×10^3^ kg/m^3^	1.40	1.38	1.34	1.54	1.25	–
Flakiness index	%	9	22	+	+	19	<35 [50]
Elongation index	%	24	23	+	+	15	<35 [50]
Acid soluble sulphate	%	0.03	0.04	0.03	0.02	0.21	<0.80 [48]
Acid soluble chloride	%	0.01	0.01	0.01	0.01	0.02	<0.05 [49]
Aggregates impact	%	28	24	+	+	24	–
LA abrasion	%	28	24	+	+	28	<40 [48]
Aggregates crushing	%	26	23	+	+	23	<45 [52]
Ten percent fines	kN	160	180	+	+	190	≥80 [48]
Moisture content	%	0.1	0.1	4.7	1.0	1.5	–
Soundness	%	1.1	2	6	+	6	<10 [48]

**Table 6:** Fresh and physical properties of concrete.

Mix Designation	Slump	Density	Porosity	Water Absorption
	mm	kg/m^3^	%	%
N/PC	200	2480	7.4	1.5
N/G50	200	2480	6.2	1.4
N/S10	200	2460	3.7	0.8
N/G50S10	210	2470	4.0	0.8
R/PC	220	2410	11.5	2.2
R/G50	200	2340	11.5	2.5
R/S10	200	2330	10.9	2.1
R/G50S10	220	2294	10.2	2.2

**Table 7:** Compressive strengths of NCA concrete series (Ft = *t*-day compressive strength determined on 150 mm cubes; ft = *t*-day compressive strength determined on 150 × 300 mm cylinders).

Mix Designation	Cube					Cylinder		Cylinder/Cube	
	F_7_ MPa	F_28_ MPa	F_56_ MPa	F_90_ MPa	F_180_ MPa	*f*_28_ MPa	*f*_90_ MPa	*f*_28_/F_28_	*f*_90_/F_90_
N/PC	59	66	66	68	72	63	67	0.95	0.99
N/G50	48	63	63	65	71	51	64	0.81	0.98
N/S10	60	84	86	87	94	83	81	0.99	0.93
N/G50S10	54	72	78	84	84	71	82	0.99	0.98

**Table 8:** Compressive strengths of RCA concrete series (Ft = *t*-day compressive strength determined on 150 mm cubes; ft = *t*-day compressive strength determined on 150 × 300 mm cylinders).

Mix Designation	Cube					Cylinder		Cylinder/Cube	
	F_7_ MPa	F_28_ MPa	F_56_ MPa	F_90_ MPa	F_180_ MPa	*f*_28_ MPa	*f*_90_ MPa	*f*_28_/F_28_	*f*_90_/F_90_
R/PC	44	53	55	59	63	41	58	0.77	0.98
R/G50	40	51	56	56	59	47	54	0.92	0.96
R/S10	41	53	55	58	58	45	54	0.85	0.93
R/G50S10	34	45	51	54	60	44	50	0.98	0.93

**Table 9:** Parameter values used for the calculation of concrete compressive strength.

Mix Designation	Parameter	
	$f_{ref}$ (MPa)	s
N/PC	65	0.18
N/G50	61	0.25
N/S10	83	0.20
N/G50S10	74	0.25
R/PC	54	0.20
R/G50	51	0.25
R/S10	52	0.23
R/G50S10	47	0.30

**Table 10:** Modulus of elasticity and flexural tensile strength results.

Mix	Modulus of Elasticity		Flexural Tensile Strength	
	(GPa)		(MPa)	
	28 Days	90 Days	28 Days	90 Days
N/PC	43.3	43.7	3.82	4.30
N/G50	43.0	42.8	3.86	4.30
N/S10	43.9	45.2	4.05	4.45
N/G50S10	46.5	46.5	4.37	4.85
R/PC	28.8	34.3	3.44	3.96
R/G50	26.9	31.1	3.95	4.27
R/S10	29.1	31.0	3.61	3.79
R/G50S10	28.0	28.2	3.51	3.66

**Table 11:** Tensile strength expressions proposed by different standards and studies.

Reference	Tensile Strength Equation	Equation Number
EC2-04 [97]	$f_{ctm}=2.12\;\ln\left(1+\left(\frac{f_{c,cy}^{\prime }}{10}\right)\right)\;for\;concrete\;grades>C50/60$ The flexural tensile strength $f_{L}$ can be computed as	(2)
	$f_{L}=\max\left[\left(1.6-\frac{h}{1000}\right)f_{ctm};\;f_{ctm}\right]$ where *h* is the beam depth (mm)	(3)
*fib* MC 2010 [110]	$f_{ctm}=2.12\;\ln\left(1+0.1\;f_{c,cy}^{\prime }\right)\;for\;concrete\;grades>C50/60$ The flexural tensile strength $f_{L}$ can be computed as	(4)
	$f_{L}=\frac{1+\alpha_{fl}\;h_{b}^{0.7}}{\alpha_{fl}\;h_{b}^{0.7}}\;f_{ctm}$ where $h_{b}$ is the beam depth (mm); $\alpha_{fl}$= 0.06	(5)
ACI 318-14 [111]	$f_{ctm}=0.62\;\sqrt{f_{c,cy}^{\prime }}$ The conversion factor from tensile strength to flexural strength is 0.67.	(6)
Xiao (2018) [20]	$f_{L}=(-0.006r+0.24){\left(f_{c,cu}^{\prime }\right)}^{2/3}$ where *r* is the replacement ratio of RCA	(7)
Kazmi et al. (2019) [109]	$f_{L}=2.2\;{f_{c,cy}^{\prime }}^{0.2}$	(8)

Note: $f_{c,cy}^{\prime }$ and $f_{c,cu}^{\prime }$ refer to the cylinder and cube compressive strengths of concrete, respectively.

**Table 12:** Embodied carbon for main constituents of concrete (kgCO2e/t).

Material	Embodied Carbon (kgCO_2_e per Tonne of Material)	
	Without Transportation in the UAE	With Transportation in the UAE
OPC	709	745
GGBS	121	157
SF	96	132
Coarse aggregate	46	82
Fine aggregate	14	50
Recycle coarse aggregate	22	58

## Data Availability

The raw data supporting the conclusions of this article will be made available by the authors on request.
